# Successful pregnancy with intracytoplasmic sperm injection after bacterial contamination of embryo culture in *in vitro* fertilization: a case report

**DOI:** 10.1186/s13256-024-04521-3

**Published:** 2024-05-15

**Authors:** Eva Berkes-Bara, Annamaria Nemes, Beata Dudas, Kata Joo, Akos Murber, Gyorgyi Fekecs, Janos Urbancsek, Peter Fancsovits

**Affiliations:** Division of Assisted Reproduction, Department of Obstetrics and Gynaecology, Semmelweis University, Baross U. 27., 1088 Budapest, Hungary

**Keywords:** Bacterial contamination, Embryo culture medium, ICSI, IVF, Bacteriospermia, *Escherichia coli*, Case report

## Abstract

**Background:**

Bacterial infection of embryo culture medium is rare but may be detrimental. The main source of embryo culture contamination is semen. Assisted reproduction centers currently lack consensus regarding the methods for preventing and managing embryo culture infection. In our recent case, a successful pregnancy was achieved with intracytoplasmic sperm injection after failed conventional *in vitro* fertilization owing to bacterial contamination.

**Case presentation:**

We present a case report of two consecutive *in vitro* fertilization–intracytoplasmic sperm injection cycles with photo and video documentation of the bacterial growth. A 36-year-old Hungarian woman and her 37-year-old Hungarian partner came to our department. They had two normal births followed by 2 years of infertility. The major causes of infertility were a closed fallopian tube and asthenozoospermia. Bacterial infection of the embryo culture medium was observed during *in vitro* fertilization and all oocytes degenerated. The source was found to be the semen. To prevent contamination, intracytoplasmic sperm injection was used for fertilization in the subsequent cycle. Intracytoplasmic bacterial proliferation was observed in one of the three fertilized eggs, but two good-quality embryos were successfully obtained. The transfer of one embryo resulted in a successful pregnancy and a healthy newborn was delivered.

**Conclusion:**

Intracytoplasmic sperm injection may be offered to couples who fail conventional *in vitro* fertilization treatment owing to bacteriospermia, as it seems to prevent infection of the embryo culture. Even if bacterial contamination appears, our case encourages us to continue treatment. Nevertheless, the development of new management guidelines for the prevention and management of bacterial contamination is essential.

**Supplementary Information:**

The online version contains supplementary material available at 10.1186/s13256-024-04521-3.

## Background

The occurrence of bacterial contamination in embryo culture medium is rare (0.23–0.68%) [1, 2], but it may have detrimental consequences such as reducing live birth rates [1], causing maternal pelvic infection [3], or epigenetic mutations in embryos [4]. Currently, there is no consensus regarding the management of bacterial contamination of embryo culture medium [5].

We present a case where conventional *in vitro* fertilization (IVF) treatment failed owing to the bacterial infection in the embryo culture medium. In the subsequent cycle, a successful pregnancy was achieved by using intracytoplasmic sperm injection (ICSI).

## Case presentation

Informed consent was obtained from the couple for this case report.

A 36-year-old Hungarian woman and her 37-year-old Hungarian partner came to our department with secondary infertility. The investigation revealed tubal and male factors (Table 1).

**Table 1:** Investigation of the infertile couple

Female partner		Male partner	
Occupation	IT specialist	Occupation	IT specialist
BMI	23	BMI	25
General anamnesis	Negative	General anamnesis	Negative
Gynecological anamnesis	Negative	Andrological examination	Intact external genitalia, no signs of inflammation
Reproductive anamnesis	Two healthy children, conceived naturally from the same marriage	Sperm test	Asthenozoospermia
Basal hormone values		Volume	5.5 ml
On cycle day 3		Sperm concentration	25 million/ml
FSH	4.5 IU/l	Total motility	28%
LH	3.0 IU/l	Progressive motility	21%
Estradiol	42 pg/ml	Normal morphology	5%
Prolactin	11.3 ng/ml	Total progressively motile sperm count	29 million
TSH	1.8 mU/l	Round cells	< 1 million/ml
Testosterone	3.4 nmol/l	Circumcision	No
SHBG	66 nmol/l		
DHEAS	6.8 μg/l	Syphilis, hepatitis, and HIV tests	Negative
On cycle day 21			
Estradiol	198 pg/ml		
Progesterone	20.4 ng/ml		
Syphilis, hepatitis, and HIV tests	Negative		
Vaginal ultrasound	Negative		
Physical examination	Negative		
Hysterosalpingography	Regular uterine cavity, open left and closed right fallopian tube		

*BMI* body mass index, *DHEAS* dehydroepiandrosterone sulfate, *FSH* follicule-stimulating hormone, *hCG* human chorionic gonadotropin, *HIV* human immunodeficiency virus, *IT* information technology, *LH* luteinizing hormone, *SHBG* sexual hormone binding hormone, *TSH* thyroid-stimulating hormone

As a first step intrauterine insemination was performed. Pregnancy was not achieved and, on the basis of the reduced sperm parameters (total progressively motile sperm count 1.1 million), we planned IVF treatment for the next cycle.

Following ovarian stimulation based on the gonadotropin-releasing hormone (GnRH) antagonist protocol, 16 oocytes were retrieved using transvaginal ultrasound-guided follicular puncture.

The male partner received written information regarding abstinence and hygiene rules, and then semen was collected by masturbation in a sterile container. Laboratory procedures were carried out using the standard protocol [6]. Semen was incubated at 37 °C. After liquefaction, the sample was homogenized and examined. The sample had a volume of 4.4 ml and a sperm concentration of 14.5 million/ml with progressive motility of 29%, without a significant number of round cells. Progressively motile spermatozoa were isolated by a combined method of density gradient centrifugation and swim-up technique. A two-layer SpermGrad (Vitrolife, Göteborg, Sweden) density gradient centrifugation was used according to the manufacturer’s instructions. Swim-up technique was applied following density gradient centrifugation to obtain a sample with high progressive motility.

After processing, the semen sample contained 1 million progressively motile spermatozoa, so we performed *in vitro* fertilization. Oocyte and embryo culture was performed in a culture media product line called “G-series” (Vitrolife) containing gentamicin.

**Fig. 1:**
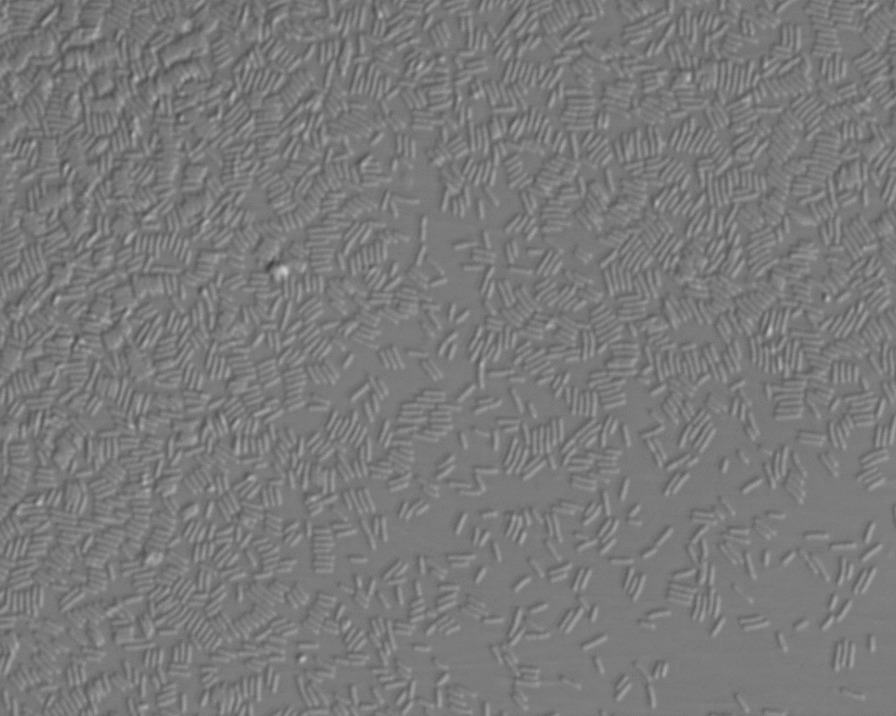
Proliferation of bacteria in the embryo culture medium after conventional *in vitro* fertilization

Following 16 h of coincubation of oocytes and sperm, a proliferation of rods was observed in the culture media and all oocytes degenerated (Fig. 1).

Samples were sent for microbiological culture. The control culture medium and cervical swab proved to be negative. The sperm suspension and the contaminated culture medium contained *Escherichia coli* (*E*. *coli*) and *Staphylococcus hominis*.

The patient was disappointed by the failed treatment, but had confidence in the department’s work.

On the basis of a thorough review of the literature, we planned ICSI treatment 3 months later. After ovarian stimulation using the antagonist protocol, 14 oocytes were retrieved by means of follicular aspiration. Semen was collected as discussed above. The volume of the semen sample was 1.8 ml and had a sperm concentration of 40.5 million/ml with progressive motility of 46%, without a significant amount of round cells. Progressively motile spermatozoa were isolated by density gradient centrifugation. After processing, the sample contained 0.3 million progressively motile spermatozoa. ICSI was performed for fertilization and embryo culture continued in an Embryoslide + group culture dish in an Embryoscope + time-lapse incubator (Vitrolife), which enables a precise evaluation of embryo morphology [7].

Out of 14 oocytes, 3 fertilized normally, 1 abnormally, and 10 were not fertilized. During pronucleus evaluation, bacterial proliferation was detected in one fertilized oocyte, which was recorded on a time-lapse video (Additional file 1. Intracellular proliferation of bacteria in a fertilized oocyte after ICSI). We did not observe any contamination in the other oocytes or culture medium. The fertilized but infected oocyte was removed from the culture plate, and its zona pellucida was opened under a microscope by using a microsurgical laser. A mass of bacteria emerged from the cell, which was captured on video (Additional file 2. Bacteria emerging from the infected oocyte after opening the zona pellucida). Microbiological analysis of the contaminated culture medium revealed *E*. *coli* in dominant germ counts and *Cutibacterium acnes*. The sperm culture also showed *E*. *coli*.

Because of the repeated positive semen cultures, the husband was referred to an andrologist, and 2 × 500 mg cefuroxime treatment was initiated.

**Fig. 2:**
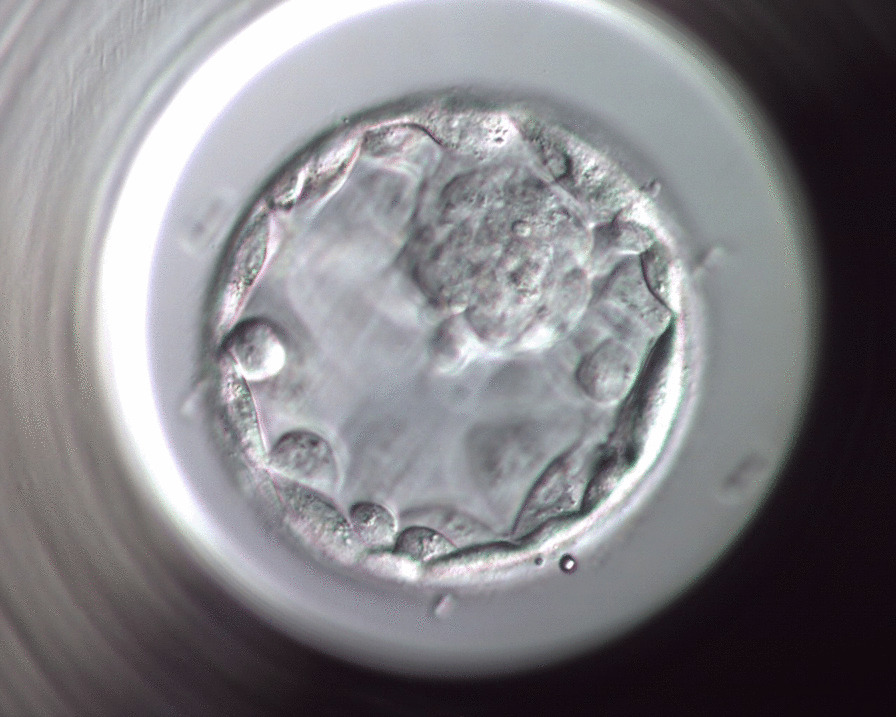
A good-quality blastocyst-stage embryo resulting in pregnancy

Two of the fertilized oocytes were not contaminated and their further culture resulted in normal embryo development and good-quality blastocysts. Cervical culture proved to be negative for bacteria, fungi, and sexually transmitted diseases (*Chamydia trachomatis*, *Neisseria gonorrheae*, *Trichomonas vaginalis*, *Mycoplasma genitalium*, and *Mycoplasma hominis* by molecular testing). Given these points, one blastocyst-stage embryo was transferred on the fifth day after fertilization (Fig. 2).

The other embryo was cryopreserved.

Progesterone (3 × 200 mg vaginal suppositories) was given to support the corpus luteum function.

After positive human chorionic gonadotropin (hCG) tests, a viable singleton pregnancy was confirmed by vaginal ultrasound at 6 weeks of gestation. The woman’s gratitude was heightened by the challenging path to pregnancy. No complications occurred during routine prenatal care, and genetic testing of the fetus confirmed normal chromosomes. In February 2023, a healthy female newborn was delivered by cesarean section at 40 weeks of pregnancy. We intend to monitor the development of the child.

## Discussion

### Bacterial contamination of the embryo culture medium

The main source of bacterial contamination of the embryo culture medium is the semen sample [8]. Bacteriospermia is defined as a bacterial count greater than 10^3^/ml [9]. It can occur in case of infection, contamination or urethral colonization, and the differentiation between these entities is challenging [10]. In addition, a further infection or chronic disease may worsen the prognosis of this condition [11]. Since our patient was symptomless and did not have abnormal physical findings or leukocytospermia, a clear diagnosis could not be established. Most researchers agree that bacteria can have a negative impact on sperm parameters [12]; however, the majority of studies demonstrated no significant difference in the effectiveness of IVF treatment in the presence or absence of bacteriospermia [13].

The poor fertilization rate, despite the use of ICSI, may be a reflection of the gametes’ quality [14].

Bacterial contamination of the culture dishes can result in the poor quality of developing embryos [2, 15]. Although direct infection and complete destruction of embryos by bacteria are unlikely, we observed total degeneration of all oocytes in our IVF cycle. After ICSI, we detected intracytoplasmic proliferation of bacteria in one of the fertilized oocytes, which may be explained by the direct injection of bacteria adhered to the spermatozoa. Tight adhesion to the sperm midpiece after centrifugation has been described with *Staphylococcus saprophyticus*, but not with *E*. *coli* [16].

As in our case, *E*. *coli* seems to be the most common cause of contamination of embryo cultures and it is usually resistant to the antibiotics used in culture media [2, 8]. *E*. *coli* can cause membrane defects and cytoplasmic vacuoles in spermatozoa [17]. These alterations affect the neck, the mid-piece, and the acrosomal membrane, which may result in immobilization and impaired acrosomal function. This hypothesis can explain the extremely poor fertilization rate we observed after ICSI.

### Prevention of contamination

To prevent microbial contamination, we strictly follow the rules provided by the World Health Organization for the retrieval and processing of semen [18].

Routine microbial culture of semen before IVF allows direct detection, but it is costly [8] and the clinical relevance of positive cases is controversial [13]. Microbiological screening of semen is not routinely performed in our department. If the number of round cells in the sample is elevated, we assess the number of white blood cells and if the number exceeds 1 million/ml, patients are referred to an expert andrologist. We observed neither bacteria nor a significant amount of round cells using microscopy and there was no sign of inflammation. Owing to repeated bacterial contamination, the patient was referred to an andrologist, who initiated antibiotic treatment. Treatment of clinically symptomatic infections is obligatory, but in cases of symptomless alteration of the normal flora, it is questionable [5]. Despite antibiotic treatment and a negative control sperm culture, recurrent bacteriospermia can still occur [19].

Comparing the technique of fertilization, contamination of the embryo culture medium seems to be more common in IVF than in ICSI treatments [1, 2, 8, 15]. A possible explanation for this is that only one single sperm is used from the semen solution during ICSI. Although the use of ICSI is expensive and it is not always beneficial in cases of non-male factor infertility [20], some working groups have suggested that it may help to avoid contamination of the culture solution [8, 15]. On the basis of this, we planned ICSI treatment after the failed IVF cycle and were able to avoid bacterial infection in the culture medium. However, we observed intracytoplasmic infection in one fertilized oocyte. To the best of our knowledge, this phenomenon has not been described in literature so far.

### Management of contamination

Regarding the management of contamination, there are only a few experimental cases available.

In a recent case report, a massive *E*. *coli* infection was detected in the embryo culture medium after ICSI; however, a healthy baby was born as a result of the treatment and the development was normal during the 2.5 year long follow-up [19]. The authors concluded that washing with gentamicin helped to remove the contamination and that freezing–devitrification might have contributed to the disappearance of the bacteria.

Shu *et al*. found that removal of the zona pellucida might help if there is still contamination after washing the embryos, as bacteria might adhere to the zona pellucida [15].

Interestingly, the three fertilized oocytes were cultured in the same well in our case, but the infection of the contaminated one remained intracellular and therefore did not affect the other two embryos. Some working groups advise against embryo transfer from contaminated culture medium [2, 21]. Du *et al*. discarded embryos if all of them were contaminated but did not contraindicate the transfer of normal embryos if there was only partial contamination [1]. Since the development of the two blastocysts was unaffected in our case, we considered the embryo transfer to be safe. This way we did not have to cancel the cycle and a healthy baby was born from the treatment.

## Conclusion

For almost 30 years of our department’s operation, we have only experienced one case of microbial infection in the IVF system. This suggests that we are following our protocols correctly when it comes to prevention. In our recent case, we observed that after conventional IVF, there was massive bacterial proliferation in the embryo culture medium, while after ICSI, bacterial infection was only detected in one fertilized oocyte. The source of the infection was found to be the semen. Although we cannot further conclude from one case, the use of ICSI may be an effective and safe procedure to prevent contamination of embryo culture medium in the case of bacteriospermia. ICSI may be offered to couples who fail conventional IVF treatment because of chronic bacterial colonization. Although this appears to be a rare event, new guidelines regarding the management of bacterial contamination of embryo culture medium are needed. A proper management of embryo culture infection would help to reduce the need for canceling cycles, while also being safe for the patient. Although cost–benefit studies would be required, our case encourages us to continue assisted reproduction treatments even if bacterial contamination appears.

### Supplementary Information

**Additional file 1.** Intracellular proliferation of bacteria in a fertilized oocyte after ICSI.

**Additional file 2.** Bacteria emerging from the infected oocyte after opening the zona pellucida.

## Data Availability

Data sharing is not applicable to this article as no datasets were generated or analyzed during the current study.

## Abbreviations

ART: Assisted reproductive technology
*E*. *coli*: *Escherichia coli*
DHEAS: Dehydroepiandrosterone sulfate
FSH: Follicle-stimulating hormone
hCG: Human chorionic gonadotropin
ICSI: Intracytoplasmic sperm injection
IVF: In vitro fertilization
LH: Luteinizing hormone
SHBG: Sexual hormone binding hormone
TSH: Thyroid-stimulating hormone
